# Improving tuberculosis care in low income countries – a qualitative study of patients' understanding of "patient support" in Nepal

**DOI:** 10.1186/1471-2458-9-190

**Published:** 2009-06-17

**Authors:** Christopher P Lewis, James N Newell

**Affiliations:** 1 University of Leeds, Faculty of Medicine and Health, Worsley Building, Leeds LS2 9JT, UK; 2 University of Leeds, Nuffield Centre for International Health and Development, Charles Thackrah Building, 101 Clarendon Road, Leeds LS2 9LJ, UK

## Abstract

**Background:**

In the new Stop TB Strategy for Tuberculosis (TB) Care, direct observation of treatment has been replaced by "supervision and patient support". However, it is still unclear what patient support means and how it is to be best implemented. The objective of this study was to accurately document patients' support needs during TB treatment from their own perspectives, to inform development of appropriate support and supervision strategies that meet patients' needs.

**Methods:**

In-depth individual interviews and focus group discussions were conducted in three districts in Nepal. Analysis took place concurrently with data collection to allow emerging issues to guide selection of subsequent interviewees. In total 23 patients, 15 male and 8 female, were interviewed and six focus group discussions were held. Issues from these interviews were grouped into emergent themes.

**Results:**

Respondents reported that the burden of treatment for TB was high, particularly in terms of difficulties with social and psychological aspects of undergoing treatment. They saw three main areas for support during their treatment: relevant information for them and their families about their disease, its treatment, potential side-effects and what they should do if side-effects arise; approachable and supportive healthcare staff with whom patients feel comfortable discussing (often non-medical) problems that arise during treatment; and some flexibility in treatment to allow essential elements of patients' lives (such as income generation, food-growing and childcare) to continue. They were anxious to ensure that family support did not absolve healthcare workers from their own support responsibilities.

**Conclusion:**

In order to support people with TB more during their treatment, health policy and practice must appreciate that TB affects all aspects of TB patients' lives. A focus on caring for each patient as an individual should underlie all aspects of treatment. Improved communication between healthcare providers and patients and increased patient knowledge and understanding of the treatment programme would give those receiving treatment a sense of individual empowerment and raise their confidence in treatment.

## Background

Tuberculosis is a profound and increasing global health problem, with 9.27 million new cases and 1.6 million deaths annually, with 1.058 million of these deaths occurring in 22 high burden countries [1].

The Stop TB Strategy, launched in 2006, replaces supervision through direct observation during treatment by one based around the provision of effective "supervision and patient support" based upon effective two-way communication between healthcare providers and those receiving treatment. [2] This patient-centred approach is also reflected in both the International Standards for TB Care (ISTC) and the Patients' Charter for TB Care. [3,4]. Increased patient empowerment may reduce the burden to patients, increase case finding and promote treatment completion. However, little operational research to document patients' perspectives on provision of effective support has been undertaken. In response, we designed a qualitative study in Nepal with the objective of identifying the support needs of TB patients from their own perspectives.

In Nepal, 63% of the population is infected with tuberculosis. Approximately 49 000 individuals develop active TB and 6400 die annually [5]. Over a third of TB patients have poor access to healthcare. Nepal is moving towards a national strategy for a more patient-centred approach to treatment, making it an appropriate environment in which to study patient support during TB treatment.

## Methods

We purposively selected three sites: Kathmandu; Kapilvastu district; and Baglung district (Table 1) to represent different types of geographical sites with varying patient demographics across Nepal. During August/September 2008, we carried out in-depth interviews to explore respondent experiences with healthcare workers during diagnosis and treatment; interaction with family and community members during treatment; illness stigma; and disease awareness and education. Following initial interviews, subsequent questions were adjusted based on emerging themes (Appendix 1). In total 23 patients (15 male and 8 female) attending their local healthcare facility were randomly selected, these numbers being chosen to reflect the 2:1 male/female ratio of TB patients in Nepal [5]. Of these patients, 15 were in the intensive phase (first 2 months) of treatment with the remainder 4 months into their treatment programme. We also conducted six FGDs, two of five women patients, two of five men patients, one of local community members, and one of local community medical assistants. In order to select patients for the FGDs, the healthcare facility register was used to anticipate when individual patients would be attending clinic: these patients were then approached and ask to form part of a group discussion to be held at the healthcare facility.

**Table 1:** General information about the three districts studied in Nepal (2006/07 data)

	**Urban Kathmandu**	**Kapilvastu District**	**Baglung District**
**Population**	1,250,000	540,000	290,000

**Geography**	Urban	Plain	Hill

**Surface Area (km^2^)**	395	1738	1784

**Number of DOTS facilities**	73	85	66

**Case finding rate (%)**	41	49	46

**Treatment success (%)**	84	84	85

During interviews and FGDs the interviewer/facilitator took notes and made audio recordings which were then transcribed, coded and grouped into emergent themes.

Approval for the research was granted by the National Research Council of Nepal and a University of Leeds ethical committee. All respondents gave written informed consent before each interview.

## Results

### 1. Patients' interactions with healthcare workers

Individual interviewees and members of the patient FGDs both voiced major concerns about a lack of individual counselling during diagnosis and treatment, and poor explanation of the treatment programme. The majority placed emphasis upon not being given information about the drug regimen and its potential side effects. The majority reported they were spoken to for less than five minutes at diagnosis. They were seldom given reasons for the long duration of treatment, or told the importance of continued adherence and treatment completion. This undermined their confidence in the treatment programme and left them reluctant to approach healthcare workers with concerns. For example, a 26 year old woman described how she knew very little about TB or its treatment. She had received no clear information on why treatment took so long, the importance of treatment adherence and completion or why her treatment was being supervised. She observed, "*I have no choice but to take the medication when and where the people here tell me. I don't understand why I have to take all these tablets every day for so long, but I have no choice, I want to get better*" (Patient 3, Kapilvastu). She had little confidence in the healthcare centre staff or her treatment, felt she had no say over her treatment and could not approach healthcare staff with her concerns for fear of recrimination.

The three individual respondents, who had received what they considered a full and appropriate explanation of their treatment felt confident in their treatment, were more willing to approach healthcare workers with concerns and felt they were active participants in their treatment.

Two interviewees reported that they were having considerable problems with loss of vision and co-ordination. They had not consulted healthcare workers because they did not realise there were potential risks (and solutions). They were adamant that they had never been told in detail about the medication they were receiving, possible side-effects, or what to do if they experienced side-effects.

The majority of respondents reported that treatment at the healthcare facility was generally well tolerated because of flexibility given to them by healthcare workers. Whilst the guidelines of the National Tuberculosis Programme state that treatment observation is to be carried out on a daily basis [6], in practice the healthcare facility often allowed some flexibility. Fifteen respondents reported that the role and purpose of daily treatment observation was not explained. Their interpretation was that they could not be trusted to manage and complete their treatment. "*The people at the clinic don't think I will take my medication when I'm supposed to ...They think we're all stupid and can't be trusted*" (Patient 2 Kapilvastu.)

Two respondents reported that they felt that being allowed daily observation by a family member rather than a health worker eliminated the constraints of facility-based supervision, but meant that they received less support from the healthcare centre. One individual had discontinued treatment for a month because of severe side effects before anyone from the healthcare centre followed up on his non-appearance for a routine check up. He commented, "*The people at the hospital think they don't have to worry about me because my family go and get my medicine for me*" (Patient 4, Baglung). Family members also described that although they were supervising their relative's adherence, they had received no education about TB and its treatment, which they felt was essential to help them to support their relative.

The majority of those interviewed both individually or as part of a group discussion from across all three districts shared concerns about lack of contact with healthcare staff and a reluctance to approach them. An individual hospitalised with suspected multi-drug resistant (MDR) TB described how in the three months he had been receiving treatment he had hardly had any contact with healthcare staff. He was very distressed: he described how he was struggling to cope with his treatment, not only because of his poor health, but also because he felt completely isolated even though he was in a hospital surrounded by healthcare workers. He commented, "*Today is the longest that anyone has spoken to me since they brought me into the hospital because I was sick*" (Patient 6, Baglung). He added that there was nobody who could support him during his time in hospital, and if he were strong enough he would return home to his family who would help him more. With his approval, we discussed with healthcare staff the possibility of transferring the patient to his home village, where he could receive the same level of medical treatment but would be in a more supportive environment with his close family. They responded, *"... the patient gets two meals a day and good medicine whilst he is here in hospital. Moving him would take a lot of effort ... the hospital in X [his home region] will just do what we are doing here*" (discussion with community medical assistant, Baglung).

### 2. Patients' interactions with family members

The majority of TB patients interviewed stated that during their treatment programme they were most likely to disclose their TB status to close family members, who in turn provided the highest level of support during treatment. The majority of respondents did however say they often felt considerable isolation, both physical and mental, due respectively to precautions taken against disease spread and because of a lack of understanding of TB. This isolation from close family members was stated as being the most difficult part of their treatment programme to deal with on a day-to-day basis. Whilst individuals receiving treatment often reported that they were motivated to complete treatment, a sense of isolation in their family home, and a lack of anyone to whom they felt they could relate were the elements that had most affected their self-esteem, made them feel most uncomfortable and made their treatment experience most difficult. *"Even people at home treat me differently, whenever I cough everyone looks at me...they are scared of me*." (Patient 4 Kathmandu.) A number of these individuals interviewed reported that it was difficult for them to speak to family members about their problems and concerns. Poor insight into TB and its treatment meant that through no direct fault of their own, family members could not provide the right type of support to those undergoing treatment. This situation was highlighted during the patient FGDs, in which participants spoke at length to each other about problems they were having at home, and described that often family members could not help them because they didn't understand their situation.

Individuals who took part in these FGDs spoke afterwards about how they felt better by just sitting and talking with people in a similar position to themselves. Individual group members stated that they could relate to other group members better than those at home because of the fact that they were all in a similar position. They said this meant that they could understand each others' problems much better. After two FGDs in Kathmandu, group members exchanged phone numbers with a view to arranging another ad-hoc meeting in the future.

Individuals receiving treatment said that they could be supported more in their home if their family were better educated about TB in general, allowing them to feel more confident in sharing some of their concerns and feelings during their treatment. Conversely, those few respondents whose close family had a general understanding of TB were more likely to share problems and concerns and reported feeling less isolated, more motivated and better understood.

### 3. Patients' interactions with the wider community

The majority of respondents believed that community understanding of TB was poor. They reported that stigma surrounding TB was high: because of this most had not disclosed their TB status to any community members for fear of discrimination towards themselves or their relatives. The hospitalised MDR patient mentioned above also described how when other TB patients heard of his TB status, they had actively separated themselves from him, speaking to him very little and had asked for his bed to be moved away from theirs. This had a profound effect on him in terms of his overall wellbeing and compounded his feelings of isolation.

In two cases, respondents hid their disease because of a fear of loss of employment if community members learnt they had TB. *"If the people who give me work find out that I have TB they won't give me any more (work)....I have to think about feeding my family and other things*" (Patient 1 Kapilvastu). Some stated that they had delayed seeking diagnosis because of fear of the consequences of a diagnosis of TB. A majority of respondents felt that high levels of TB stigma were due to a fundamental lack of understanding about TB. Healthcare workers reported that patients are often very ill at the time of diagnosis because of an initial reluctance to seek treatment. *"It is too long (before some patients come for treatment)...they wait too long, this means that treating them is very difficult" *(Community Medical Assistant, Kapilvastu). Healthcare personnel felt this delay was due to a lack of awareness of TB and also a fear of community discrimination. Community healthcare workers said that this delay was most marked in women due to the local culture, commitments at home and very poor awareness with regard to TB. This was a situation confirmed by individual interviews with those undergoing treatment including a number of female interviewees.

## Discussion

Our interviews show that a dichotomy exists between the interpretations by patients and healthcare providers of "supervision and patient support."

In order to better support people with TB during their treatment, the healthcare system and its employees must appreciate that TB disease affects all aspects of individuals' lives, and must provide treatment programmes which recognise this. A focus by the healthcare system and its employees on a biomedical model of treatment, concentrating primarily on accurate diagnosis and provision of curative medicines, may show high levels of treatment success as measured by routine programme statistics, but this success is often at a considerable cost to patients. Study participants report that the burden of treatment for TB is high, particularly in terms of difficulties with social and psychological aspects of undergoing treatment. Whilst the Stop TB strategy requires that these aspects are addressed in a more patient-centred approach to TB care, they are not addressed or fully appreciated by the current approach to TB control in Nepal, although there is now movement towards a national strategy for a more patient-centred approach.

Informal flexibility in treatment supervision is welcomed by patients and has meant that a significant barrier to seeking and continuing with effective TB treatment is reduced. The results also support evidence provided by studies in other low-income countries that by incorporating a degree of flexibility in treatment supervision, one of the significant barriers to effective TB care can be removed [6]. The feasibility of incorporating this approach into treatment policy in Nepal, whilst ensuring that it does not allow healthcare providers to compromise on patient care, should be explored.

Our findings support current literature which reports that health personnel involved in TB care are lacking in basic communication skills [7]. Effective communication and the establishment of good working relationships between those with TB and healthcare providers, allowing patients to feel they have an active role to play in their treatment as encouraged in the Stop TB guidelines, should underlie TB healthcare provision. This is supported by research which shows that changing professional practice, leading to improved quality of professional-patient interactions results in an improvement in treatment adherence and completion [8,9].

At diagnosis, an extended period of consultation with more information being provided regarding TB and its treatment would begin to establish a firmer relationship between service users and providers. Discussion about the medication the newly diagnosed individual is to receive, an explanation of supervision and the importance of continued adherence would reassure patients and improve their confidence in the TB treatment programme. Improved awareness and understanding would also increase the sense of empowerment that individuals with TB feel in their roles as active participants in the treatment process.

Those receiving treatment often feel that they currently cannot approach healthcare workers with their concerns and problems during treatment. Establishing a better working relationship, in which the patient feels s/he is an active participant, would help to improve this situation. It would mean that problems which may arise during treatment as a result of medication side-effects or other unrelated issues would not go unanswered, as is currently often the case. This is supported by studies in other low-income countries which show that where those receiving treatment have an improved understanding of their drug regimen, they default less, and are more likely to approach healthcare workers if problems arise [10].

Looking to close family members for support during treatment is not unique to those in Nepal [11]. Promoting increased knowledge and awareness of a disease and its treatment within a patient's family should form a key part of effective healthcare policy for treatment of TB, as well as other communicable diseases. A better understanding of TB and its treatment within the families of those undergoing treatment would allow both parties to feel that they could share their concerns and problems more openly. This would help to remove the sense of isolation from close family which respondents reported as being the most difficult aspect of their treatment. The intensive phase of treatment is the time when those receiving treatment require most support. An opportunity for those receiving treatment and close family members to receive counselling together in consultation with a healthcare worker following diagnosis during this time would allow both these individuals to receive, and families to provide, more support during the treatment programme.

In those cases where patient support is to be family-based, healthcare staff should not view this arrangement as one which absolves them of responsibility to an individual with TB. For family based DOT to be successful requires that the healthcare centre and staff supervise and support the family members responsible for supporting the individual undergoing treatment. Provision of counselling both to those receiving treatment and those who are to be supporting treatment is essential to ensure appropriate support. This would not only improve confidence in treatment but also allow treatment to become a partnership between the patient and family members, enabling a higher level of support, and improving the treatment experience of all concerned.

Individuals receiving TB treatment say that they view a lack of understanding of TB within both their family and the wider community as a major reason why the treatment period is one characterised by a distinct sense of isolation. Providing a support network during treatment which gives those undergoing treatment the opportunity to speak to other patients currently receiving treatment is one way that patients feel this sense of isolation can be reduced. These groups could be established without placing significant increased demand on the healthcare centre or its staff in terms of time or resources. This approach has been documented in the literature as improving the treatment experience for those receiving TB treatment [12]. The feasibility of a similar approach in Nepal warrants further investigation.

Our findings add to the evidence which highlights issues of stigma, poor understanding and awareness of TB and problems with effective service provision as being major reasons why many people with TB delay seeking TB diagnosis and treatment, affecting their treatment prognosis as a result [13,14]. Interviews with TB patients and wider community members show that it is healthcare workers that people look towards to raise understanding and awareness of TB. Investing more time in providing patients with full and clear explanations about TB and its treatment has been documented as being an effective method of raising patients' levels of motivation and promoting treatment adherence [15]. This research supports these findings, adding that where proper information is not given, patient confidence in their treatment programme and by extension the healthcare centre and its staff is low.

## Conclusion

In order to support people with TB more during their treatment, health policy and practice must appreciate that TB affects all aspects of TB patients' lives. A focus on caring for each patient as an individual should underlie all aspects of treatment. We should ensure that all health workers appreciate the difference between "TB patients" and "people with TB", and its implications. It is important that the healthcare system and its staff recognise that improving a person's health requires more than just the provision of effective medical treatment. Training healthcare workers in basic communication skills to promote improved interaction between healthcare providers and patients would result in a significant increase in patient knowledge and understanding of their TB treatment programme. It would give those receiving treatment for TB a sense of individual empowerment, raising their confidence in themselves and their treatment and significantly improving their treatment experience as a result.

## Competing interests

The authors declare that they have no competing interests.

## Authors' contributions

CPL developed the research proposal, data collection, analysis and manuscript draft and final production. JNN participated in designing the study, project coordination and helped to complete the final manuscript. Both authors have read and approved the final manuscript.

## Appendices

### Appendix 1

#### TB Patients Questions

*The following questions were used as a guide during patient interviews. The questions were used for guidance of conversation and are not exhaustive*.

##### Thoughts and Behaviours

• Can you describe to me what you think TB is?

• What do you understand about TB?

• Where did you learn about TB?

• What do you think causes TB? How is it spread and what is the best way to treat it?

• How is TB understood in your community?

• Do patients with TB receive different treatment from members of their family/community than those who don't have the illness?

• Does TB stigma affect your motivation to seek treatment?

• Do people in your community seek private treatment: if so why?

##### Community and Family stigma

• Do you think that TB is well understood by your community and family?

• Can you describe how your family/friends/community/employer reacted when you were diagnosed with TB?

• Can you describe how well TB and its treatment are understood in your family/community?

• Who do you think might be in a good position to help your community understand TB and its treatment better?

##### TB Education

• Can you describe what you were told about TB when you came to the healthcare centre? Did the centre teach you about the disease: if so how?

• Can you describe how you were taught about TB at the healthcare centre?

• What do you feel could be done to help patients understand their TB treatment better?

##### TB Medication

• Can you describe to me how you were taught about your medication which will help to treat your illness?

• Have you had any problems with your medication?

• Can you describe to me if you were taught about some of the side-effects that you might get from your medication and how you might be able to reduce them?

• Do you understand what side-effects are serious and when you should return to the healthcare centre?

##### TB Treatment

• Please describe for me your TB treatment programme.

• Please describe some of the difficulties that you have had while you have been receiving treatment.

• Please describe how you were told about your treatment.

• Do you understand why treatment is so long?

• Do you understand why it is important to take your treatment even when you feel better?

• Do you understand the benefits to the health of your family and community that will happen if you complete your treatment; if not would this change your attitude to treatment?

• Do you talk to other members of your family/community about your treatment; do they understand your treatment?

• What changes would make your treatment experience better?

##### Motivation

• Do you think you will be able to complete your treatment which will last for 6 months? Why?

• Can you describe the support that you have received during your illness and as you have started getting treatment from your family/community/healthcare centre?

• What do you think would make your long treatment programme easier for you?

• If patients were given something for completing their treatment that would help people to finish their treatment?

##### Supervision

• Why do you think that your treatment is supervised?

• Do you think that this supervision helps you to continue with your treatment?

• Do you feel you have any say about your treatment?

• Have you ever talked to the healthcare centre about some of your difficulties? How do they react?

• What changes would make your treatment experience better?

• How would you like to be treated: the NTP says that you must be supervised in some way, how do you think this might be done?

##### Family Support

• Please describe to me the support you have received from you family during your treatment

• Do you think that the attitude of your family has changed since you were diagnosed with TB

##### Healthcare Centre Support

• Please describe to me the support you received from the healthcare centre when you were first diagnosed. How did they help you?

• What do you think the healthcare centre could do to help you more?

• Can you describe to me how you are treated when you come to the centre each time?

• Can you describe how the centre supports you to continue with your treatment?

##### Community Support

• Please describe to me the support you get from your community. How do they help you with your treatment?

• When somebody is diagnosed with TB, does the attitude of your family/community change towards them? Why?

• Do you feel you are treated differently by your community now that you are getting your treatment?

• Who in your community is well respected? Who do people look up to for help when they need support?

## Pre-publication history

The pre-publication history for this paper can be accessed here:


